# Temporal Trends of Intracranial Hemorrhage Among Immune Thrombocytopenia Hospitalizations in the United States

**DOI:** 10.7759/cureus.9427

**Published:** 2020-07-27

**Authors:** Parth Bhatt, Priyank J Yagnik, Marian Ayensu, Abdul Wasay Khan, Abigail Adjei, Narendrasinh Parmar, Kuhoo Bhal, Keyur Donda, Fredrick Dapaah-Siakwan, Neel S Bhatt

**Affiliations:** 1 Pediatrics, United Hospital Center, Bridgeport, USA; 2 Pediatrics, University of Kansas School of Medicine, Wichita, USA; 3 Pediatrics, The Trust Hospital, Accra, GHA; 4 Pediatrics, University of Kansas School of Medicine Wichita, Wesley Medical Center, Wichita, USA; 5 Pediatrics, University of Texas Health Science Center, Houston, USA; 6 Pediatrics, Brookdale University Hospital and Medical Center, Brooklyn, USA; 7 Pediatrics, University of Maryland, College Park, USA; 8 Pediatrics, University of South Florida, Tampa, USA; 9 Pediatrics, Valley Children’s Healthcare, Madera, USA; 10 Pediatric Hematology and Oncology, University of Washington School of Medicine, Seattle, USA

**Keywords:** intracranial hemorrhage, immune thrombocytopenia, incidence, length of stay, cost of hospitalization, trends

## Abstract

Background: Intracranial hemorrhage (ICH) is a rare but severe complication in patients with immune thrombocytopenia (ITP). We aimed to examine the incidence and outcomes of ICH among ITP hospitalizations and factors associated with it. Additionally, we studied resource utilization for these hospitalizations.

Methods: Using National (Nationwide) Inpatient Sample, International Classification of Diseases, Ninth and Tenth Revision, Clinical Modification (ICD-9-CM/ICD-10-CM) codes, we studied ITP hospitalizations with occurrence of ICH between 2007 and 2016.

Result: Out of 348,906 weighted ITP hospitalizations, ICH occurred in 3,408 encounters (incidence 1.1 ± 0.04%). The incidence remained stable over time (2007-2008: 1.01%, 2015-2016: 1.20%; P = 0.3). People with age ≥25 years, especially those aged ≥65 years (odds ratio [OR] 3.69, 95% confidence interval [CI] 2.34-5.84), or those with gastrointestinal bleed (OR 1.60, 95% CI 1.18-2.16) were significantly more likely to develop ICH. Female gender (OR 0.81, 95% CI 0.68-0.97) had lower odds for developing ICH. Overall mortality in ITP hospitalizations with ICH was 26.7%. Length of stay (LOS) was longer (4.8 vs. 2.6 days) and costs of hospitalization (COH) were higher ($20,081 vs. $8,355) in ICH hospitalizations compared to non-ICH ITP hospitalizations. Increasing age and comorbidities such as gastrointestinal bleed, hematuria, and other bleeding were also associated with longer LOS and higher COH.

Conclusion: Although rare, ICH in ITP was associated with a high mortality and increased resource utilization. Clinicians should be cognizant of factors associated with risk of ICH in ITP, and future studies should reassess the ICH trends to study the impact of novel therapeutic options such as thrombopoietin receptor agonists.

## Introduction

Immune thrombocytopenia (ITP) is an acquired immune-mediated disorder characterized by isolated thrombocytopenia (platelet count less than 100 x 10^9^/liter), and an increase in the risk of bleeding due to increased destruction or decreased production of platelets [1]. ITP is known to affect both adults and children with an occurrence of 2-5/100,000 children per year and 3.3/100,000 adults per year [2]. Patients present with varying degree of bleeding ranging from petechiae, bruises, epistaxis, and gingival bleeding to visceral bleeding such as hematuria, hematochezia, and hematemesis, which is uncommon [3].

Intracranial hemorrhage (ICH), although rare, is a serious complication associated with ITP. Evidence indicates that the risk of ICH among adults with ITP is approximately 0.9%-2.1% in adults and 0.2%-0.7% in children [4]. Mortality associated with ICH in ITP patients ranges from 9.7% to 44% depending on the population studied [5,6]. Majority of prior reports on ICH have been either single center studies or limited by small sample size [7,8]. A few studies have used large administrative databases to assess pediatric and adult ITP hospitalizations; however, they were not primarily focused on ICH as an outcome and therefore lacked details of ICH incidence and outcomes trends and factors associated with it [5,6,9].

To address the knowledge gap, our study aimed to assess the trends of ITP-related hospitalizations with a diagnosis of ICH in the United States (US) from 2007 through 2016. Additionally, we aimed to study the factors associated with the incidence of ICH, mortality, and resource utilization in ITP hospitalizations with a diagnosis of ICH.

## Materials and methods

Data source

Our study cohort was derived from National (Nationwide) Inpatient Sample (NIS) database which is a large publicly available all-payer inpatient care database in the United States. NIS is part of Healthcare Cost and Utilization Project (HCUP), sponsored by the Agency for Healthcare Research and Quality (AHRQ) [10]. NIS database includes clinical and resource utilization information derived from discharge abstracts. Each hospitalization in the database is de-identified and recorded as a unique entry with one primary discharge diagnosis, up to 30 secondary diagnoses along with up to 15 codes for procedures that occurred during the hospitalization. The 2016 NIS sampling frame includes data from 46 states and the District of Columbia, covering more than 97% of the US population and including almost 96% of discharges from the US community hospitals [11]. Large sample size enables analyses of rare conditions, uncommon treatments, and special patient populations. NIS has been previously used to study trends in hospitalization in various neonatal, pediatric, and adult populations [12,13].

Study population

**Figure 1:**
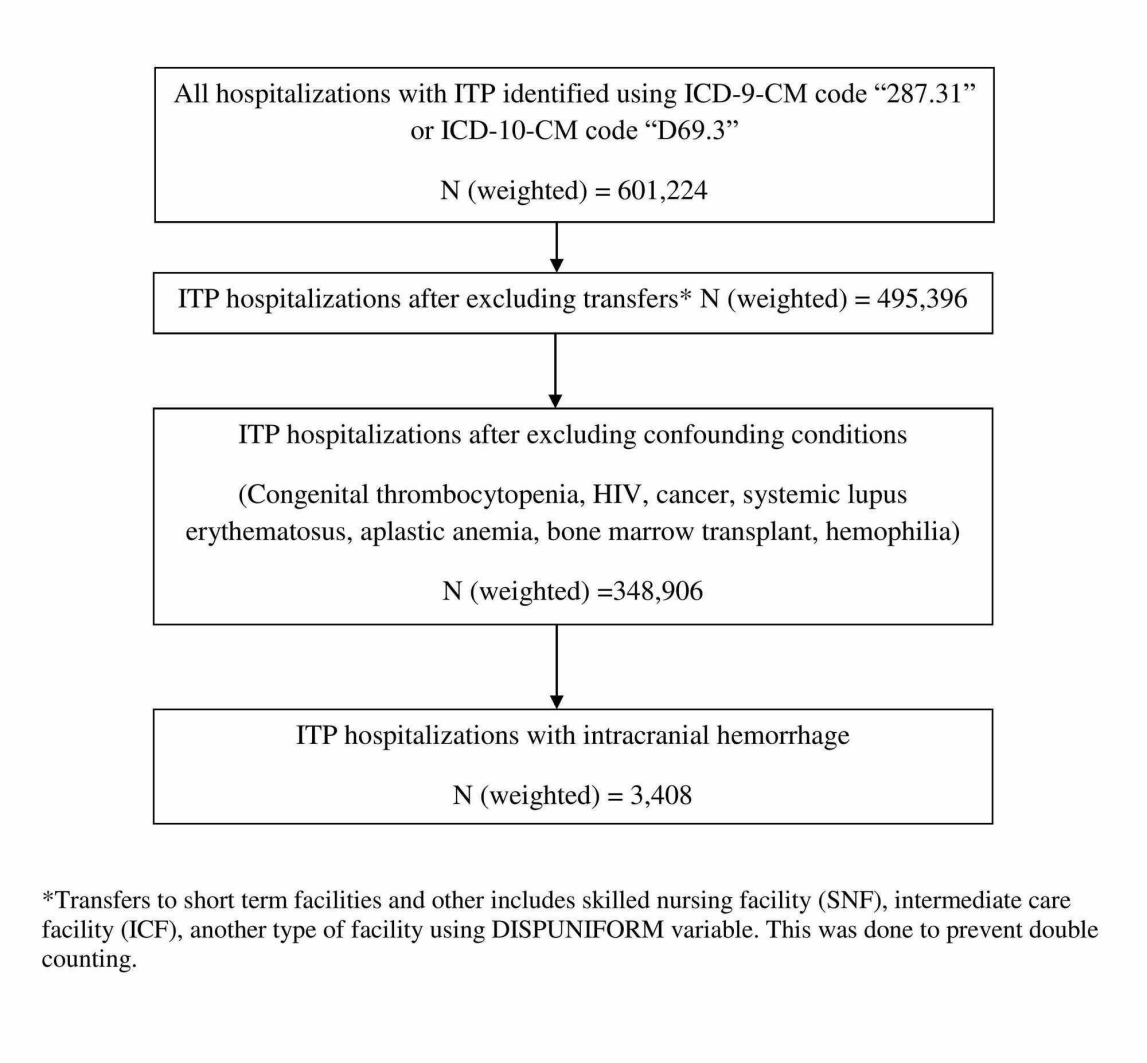
Population derivation ICD-9-CM/ICD-10-CM, International Classification of Diseases, Ninth and Tenth Revision, Clinical Modification; ITP, immune thrombocytopenic purpura; HIV, human immunodeficiency virus

We searched NIS database between 2007 and 2016 and identified all pediatric and adult ITP hospitalizations using International Classification of Diseases, Ninth and Tenth Revision, Clinical Modification (ICD-9-CM/ICD-10-CM) diagnosis codes “287.0” and “D69.3” in any of the diagnosis fields, respectively. Hospitalizations with conditions which could be confounded with ITP were identified using relevant ICD-9/10-CM codes and excluded. Similar methodology has been used in past [14]. To eliminate double counting and improve completeness of data, transfers to skilled nursing facility (SNF), intermediate care facility (ICF), another type of facility, or to short-term facility were excluded using the “DISPUNIFORM” variable [15]. Hospitalizations with ICH were identified by using ICD-9/10-CM codes. Details of population derivation are shown in Figure 1.

Definition of variables

Encounter-level baseline characteristics were studied. Patient-level characteristics such as age in years (0-14, 15-24, 25-64, and ≥65), gender, race (white, black, Hispanic, and others), median household income category in quartiles based on zip codes, primary payer (Medicare, Medicaid, private, self-pay/no charges/others), and admission type (elective and non-elective) were also studied. Additional clinical variables were identified based on relevant ICD-9/10-CM codes. Hospital-related characteristics were identified, such as hospital bed size (small, medium, and large), region (Northeast, Midwest, South, and West), location, and teaching status (rural, urban non-teaching, and teaching hospitals). NIS contains data on cost stored as total charges for each hospitalization. We utilized HCUP cost-to-charge files to estimate the cost of in-patient resource utilization. Costs reflect the actual expenses incurred in the production of hospital services, such as wages, supplies, and utility costs; charges represent the amount a hospital billed for the case [16]. Adjusted cost for each year was calculated in terms of 2016 cost after adjusting for inflation as per consumer price index data released by the US government [17]. This enabled us to standardize cost for the study period.

Statistical analysis

Baseline characteristics of ITP hospitalizations grouped by ICH status were reported using descriptive statistics. For continuous variables, median and interquartile ranges (IQR) were reported as data were found to have non-normal distribution. Categorical variables were reported as percentages. To assess differences for the univariate analysis, the chi-square test was used for categorical variables and the Wilcoxon rank-sum test for continuous variables. NIS includes sampling weights derived by stratifying NIS hospitals on variables used to generate the sample [18]. These weights were used to generate national estimates. Beginning 2012, the NIS underwent changes in sampling of hospital discharges. To account for the changes in the NIS database design, TRENDWT was used for trend analysis [19]. For trend analysis, the chi-square test of trend for proportions was used using the Cochrane-Armitage test. NIS has a complex survey design wherein discharges are clustered within the hospitals. Survey regression was used to analyze trends for continuous variables such as length of stay (LOS) and cost of hospitalization (COH). Two-level hierarchical models (smaller unit factors [encounters] nested within larger unit factors [hospital]) were created, with the unique hospital identification number incorporated as a random effect within the model. Hospital identification was incorporated as a random effect in the model to account for the impact of hospital clustering to account for possibly similar outcomes in patients being treated at the same hospital due to certain processes of care received [20]. Hierarchical regression was utilized to analyze factors associated with LOS and COH. Survey logistic regression was utilized to analyze factors associated with ICH among ITP and mortality among ICH hospitalizations. Statistical Analysis System (SAS) software v9.4 (SAS Institute Inc., Cary, NC) was used for all analyses. Two-tailed P-value of <0.05 was considered as significant.

## Results

Patient population

**Figure 2:**
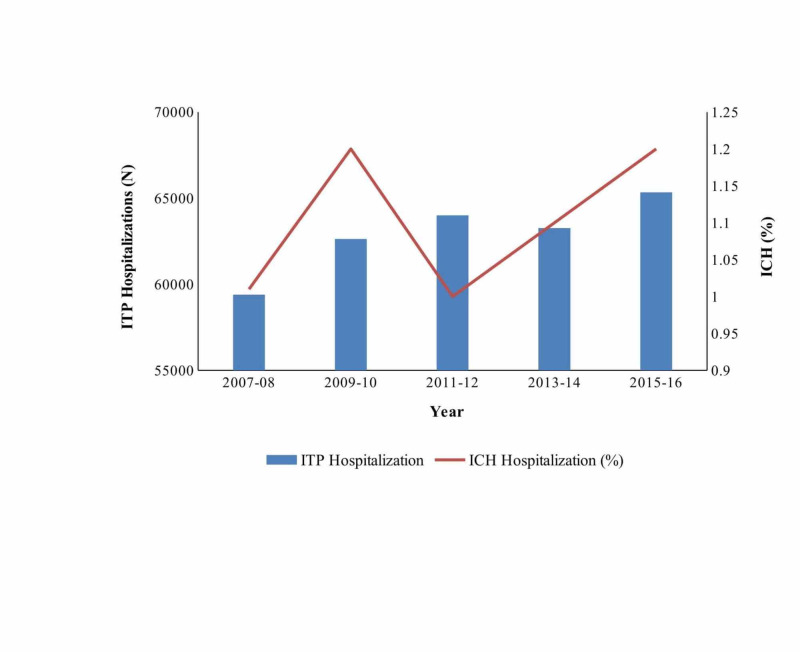
Trend of hospitalization of intracranial hemorrhage and immune thrombocytopenic purpura from 2007 to 2016 ITP, immune thrombocytopenic purpura; ICH, intracranial hemorrhage.

Among 348,906 hospitalizations with ITP over the period of 2007 through 2016, there were 3,408 hospitalizations for ICH (Figure 2).

Among ITP hospitalizations, those with ICH were older (63.4 vs. 51.6 years, P < 0.001) and were more likely to have a non-elective admission (92.4% vs. 78.1%, P < 0.001), and other comorbidities, such as gastrointestinal (GI) bleeding (7.7% vs. 4.3%, P < 0.001), hematuria (3.1% vs. 2.1%, P < 0.001), and epistaxis (4.8% vs. 3.6%, P < 0.001) (Table 1). There were no statistically significant differences between ITP hospitalizations with and without ICH relative to race distribution and median household income. Compared to those without ICH, ITP hospitalizations with ICH resulted in significantly higher mortality (26.7% vs. 3.2%, P < 0.001), longer LOS (4.8 vs. 2.6 days, P < 0.001), and increased COH ($20,081 vs $8,335, P < 0.001). Diringer and Edwards showed that admission of ICH patients in dedicated neurointensive care unit is associated with lower mortality compared to general intensive care unit [21]. Our finding showed that most of the ICH hospitalizations occurred in large (71.4%) and urban teaching hospitals (70.7%) and in the Southern (36.6%) and Western region of the US (24.8%).

**Table 1:** Baseline characteristics of patients with immune thrombocytopenia hospitalized between 2007 and 2016 based on occurrence of ICH. IQR, interquartile range; ICH, intracranial hemorrhage; GI, gastrointestinal.

	No ICH	ICH	Total	P-value
ITP hospitalization, n (unweighted)	71,303	704	72,007	
ITP hospitalization, N (weighted)	345,498	3,408	348,906	
Patient-level variables				
Age (years), median (IQR)	51.6 (28.6-70.0)	63.4 (47.1-76.7)	51.7 (28.7-70.1)	<0.001
Age groups (years, %)				
0-14	10.2	5.0	10.1	
15-24	8.6	5.6	8.6	
25-64	47.8	41.3	47.8	
≥65	32.1	47.0	32.3	
Missing	1.3	1.2	1.3	
Gender (%)				<0.001
Male	39.6	47.5	39.7	
Female	60.1	52.4	60.0	
Missing	0.3	0.2	0.3	
Race (%)				
White	59.8	59.4	59.8	
Black	10.5	9.8	10.5	
Hispanic	12.2	10.8	12.2	
Other	6.6	9.2	6.7	
Missing	10.9	10.9	10.9	
Median household income category for zip code (%)				<0.001
0-25th percentile	25.7	23.1	25.6	
26-50th percentile	24.7	25.5	24.7	
51-75th percentile	24.0	23.6	24.0	
76-100th percentile	23.7	26.0	23.7	
Missing	2.0	1.9	2.0	
Primary payer (%)				<0.001
Medicare	36.8	49.3	37.0	
Medicaid	17.9	15.1	17.9	
Private	37.4	26.8	37.3	
Self-pay/no charge/other	7.8	8.5	7.8	
Missing	0.2	0.3	0.2	
Type of admission				<0.001
Non-elective	78.1	92.4	78.3	
Elective	21.5	7.7	21.4	
Hospital characteristics				
Hospital bed size (%)				<0.001
Small	11.6	6.5	11.5	
Medium	25.0	21.2	25.0	
Large	62.9	71.4	63.0	
Missing	0.6	0.9	0.6	
Hospital region (%)				<0.001
Northeast	21.1	18.4	21.1	
Midwest	22.1	20.2	22.1	
South	36.7	36.6	36.7	
West	20.0	24.8	20.1	
Hospital location and teaching status (%)				<0.001
Rural	7.5	3.8	7.4	
Urban non-teaching	32.5	24.8	32.4	
Urban teaching	59.5	70.7	59.6	
Missing	0.6	0.9	0.6	
Bleeding events (%)				
GI bleed	4.3	7.7	4.3	<0.001
Hematuria	2.1	3.1	2.1	<0.001
Female-specific bleeding	0.3	0.0	0.3	0.001
Pregnancy-related bleeding	0.7	0.0	0.7	<0.001
Hemarthrosis	0.0	0.0	0.0	0.3
Hemoglobinuria	0.0	0.0	0.0	0.6
Epistaxis	3.6	4.8	3.6	<0.001
Other bleeds	3.1	5.4	3.1	<0.001
Mortality (%)	3.2	26.7	3.5	<0.001
Length of stay (days), median (IQR)	2.6 (1.4-4.8)	4.8 (2.4-10.1)	2.6 (1.4-4.8)	<0.001
Cost of hospitalization ($), median (IQR)	8,355 (4,538, 16,073)	20,081 (9,536, 41,096)	8,426 (4,559, 16,229)	<0.001

Incidence of ICH

Table 2 shows the overall incidence and trends in the incidence of ICH among ITP hospitalizations according to age, sex, race, and census region in the US from 2007 to 2016. Overall, the incidence of ICH was 1.1 (±0.04) in all ITP hospitalizations. The incidence was 1.1% and 0.7% among adult and pediatric hospitalizations, respectively. There was no significant change in incidence during the study period (P = 0.3). However, the incidence declined in those aged ≤24 years (0-14 years: 0.7% in 2007-2008 to 0.4% in 2015-2016; 15-24 years: 0.8% in 2007-2008 to 0.4% in 2015-2016). On the contrary, the ICH incidence significantly increased in those aged ≥65 years, 1.5% (2007-2008) to 1.9% (2015-2016). Similarly, females (0.9% to 1.1%) and hospitalizations in the Northeast region of the US (0.9% to 1.1%) were noted to have increasing ICH trend from 2007-2008 to 2015-2016. There were no significant trends among major race/ethnicity groups. 

**Table 2:** Trends of ICH among ITP hospitalizations by age groups, gender, race and hospital region. SE, standard error; IQR, interquartile range; ICH, intracranial hemorrhage; ITP, immune thrombocytopenia.

	2007-2008	2009-2010	2011-2012	2013-2014	2015-2016	Total	P-trend
Overall ICH incidence (%, SE)	1.0 ± 0.1	1.2 ± 0.1	1.0 ± 0.1	1.1 ± 0.1	1.2 ± 0.1	1.1 ± 0.04	0.3
ICH incidence children (%, SE)	0.8 ± 0.3	0.7 ± 0.2	0.9 ± 0.2	0.5 ± 0.2	0.5 ± 0.2	0.7 ± 0.1	0.001
ICH incidence adult (%, SE)	1.0 ± 0.1	1.3 ± 0.1	1.0 ± 0.1	1.2 ± 0.1	1.3 ± 0.1	1.1 ± 0.1	0.002
Age (%, SE)							
0-14 years	0.7 ± 0.3	0.6 ± 0.2	0.7 ± 0.3	0.2 ± 0.1	0.4 ± 0.2	0.5 ± 0.1	<0.001
15-24 years	0.8 ± 0.3	0.7 ± 0.3	0.8 ± 0.3	0.8 ± 0.3	0.4 ± 0.2	0.7 ± 0.1	0.03
25-64 years	0.8 ± 0.2	1.2 ± 0.2	0.8 ± 0.1	1.0 ± 0.2	0.9 ± 0.1	1.0 ± 0.1	0.6
≥ 65 years	1.5 ± 0.2	1.5 ± 0.2	1.2 ± 0.2	1.7 ± 0.2	1.9 ± 0.2	1.6 ± 0.1	<0.001
Gender (%, SE)							
Male	1.2 ± 0.2	1.4 ± 0.2	1.2 ± 0.2	1.4 ± 0.2	1.3 ± 0.2	1.2 ± 0.1	0.4
Female	0.9 ± 0.1	1.0 ± 0.1	0.7 ± 0.1	1.0 ± 0.1	1.1 ± 0.1	1.0 ± 0.1	0.03
Race (%, SE)							
White	1.1 ± 0.1	1.2 ± 0.1	0.9 ± 0.1	1.1 ± 0.1	1.1 ± 0.1	1.1 ± 0.1	0.8
Black	1.0 ± 0.3	1.0 ± 0.3	1.0 ± 0.3	1.0 ± 0.2	1.1 ± 0.3	1.0 ± 0.1	0.6
Hispanic	1.1 ± 0.3	0.8 ± 0.2	1.1 ± 0.3	0.9 ± 0.2	0.8 ± 0.3	1.0 ± 0.1	0.2
Other	1.2 ± 0.6	1.5 ± 0.5	0.7 ± 0.3	1.5 ± 0.4	2.4 ± 0.5	1.5 ± 0.2	<0.001
Hospital region (%, SE)							
Northeast	0.9 ± 0.2	0.7 ± 0.2	0.9 ± 0.2	1.2 ± 0.2	1.1 ± 0.2	0.9 ± 0.1	<0.001
Midwest	0.9 ± 0.2	1.3 ± 0.2	0.6 ± 0.1	1.2 ± 0.2	1.0 ± 0.2	1.0 ± 0.1	0.7
South	1.0 ± 0.1	1.2 ± 0.2	0.9 ± 0.1	1.1 ± 0.2	1.2 ± 0.2	1.1 ± 0.1	0.4
West	1.3 ± 0.3	1.5 ± 0.3	1.3 ± 0.2	1.2 ± 0.2	1.4 ± 0.2	1.3 ± 0.1	0.8

Factors associated with the occurrence of ICH

Table 3 shows results of a multivariable regression analysis assessing factors associated with the occurrence of ICH during ITP hospitalizations. Age ≥25 years was significantly associated with the higher occurrence of ICH (25-64 years: odds ratio [OR] 2.23, 95% confidence interval [CI] 1.51-3.31; ≥65 years: OR 3.69, 95% CI 2.34-5.84). Comorbidities such as GI bleed (OR 1.6, 95% CI 1.18-2.16) and other bleeding (OR 1.69, 95% CI 1.19-2.42) were also associated with higher likelihood of ICH. Hospitalization in the Western census region (OR 1.62, 95% CI 1.26-2.08) and hospitalization in the medium (OR 1.64, 95% CI 1.08-2.47), large (OR 2.42, 95% CI 1.65-3.55), and urban teaching hospitals (OR 2.73, 95% CI 1.80-4.13) were associated with increased odds of ICH. Female gender was associated with less likelihood of ICH occurrence (OR 0.81, 95% CI 0.68-0.97).

**Table 3:** Factors associated with occurrence of intracranial hemorrhage during immune thrombocytopenia hospitalizations.

Variable	Odds ratio	Confidence interval		P-value
		Lower limit	Upper limit	
Age in years				
0-14	Reference			
15-24	1.27	0.77	2.10	0.4
25-64	2.23	1.51	3.31	<0.001
≥65	3.69	2.34	5.84	<0.001
Year	0.99	0.93	1.06	0.8
Gender				
Male	Reference			
Female	0.81	0.68	0.97	0.02
Primary payer				
Medicare	Reference			
Medicaid	1.01	0.72	1.41	1.0
Private	0.81	0.61	1.08	0.2
Self-pay/no charge/other	1.15	0.79	1.67	0.5
Gastrointestinal bleed	1.60	1.18	2.16	0.002
Hematuria	1.32	0.83	2.08	0.2
Epistaxis	1.35	0.92	1.98	0.1
Other bleed	1.69	1.19	2.42	0.004
Hospital region				
Northeast	Reference			
Midwest	1.02	0.74	1.39	0.9
South	1.23	0.99	1.53	0.1
West	1.62	1.26	2.08	<0.001
Hospital bed size				
Small	Reference			
Medium	1.64	1.08	2.47	0.02
Large	2.42	1.65	3.55	<0.001
Hospital location and teaching status				
Rural	Reference			
Urban non-teaching	1.43	0.92	2.20	0.1
Urban teaching	2.73	1.80	4.13	<0.001

Factors associated with mortality in ITP hospitalizations with ICH

There was no significant change in the mortality rate across the study time-period (OR 0.98, 95% CI 0.94-1.01). Table 4 shows the multivariable regression analysis assessing factors associated with mortality among ITP hospitalizations with ICH. Again, age ≥25 years, GI bleed (OR 3.13, 95% CI 2.72-3.60), and hospitalization in large (OR 1.17, 95% CI 1.00-1.37) and urban teaching hospitals (OR 1.23, 95% CI 1.02-1.48) were associated with increased odds of in-hospital mortality. Female gender was associated with decreased odds of in-hospital mortality (OR 0.76, 95% CI 0.9-0.83).

**Table 4:** Factors associated with mortality among Immune thrombocytopenia hospitalizations with intracranial hemorrhage.

Variable	Odds ratio	Confidence interval		P-value
		Lower limit	Upper limit	
Age in years				
0-14	Reference			
15-24	0.99	0.49	2.02	1.0
25-64	6.73	4.11	11.03	<0.001
≥65	16.94	10.14	28.30	<0.001
Year	0.98	0.94	1.01	0.2
Gender				
Male	Reference			
Female	0.76	0.69	0.83	<0.001
Primary payer				
Medicare	Reference			
Medicaid	0.70	0.56	0.88	0.02
Private	0.45	0.38	0.55	<0.001
Self-pay/no charge/other	0.71	0.55	0.92	0.009
Gastrointestinal bleed	3.13	2.72	3.60	<0.001
Hematuria	1.10	0.83	1.46	0.5
Epistaxis	0.70	0.52	0.95	0.02
Other bleed	1.66	1.31	2.10	<0.001
Hospital region				
Northeast	Reference			
Midwest	0.89	0.76	1.04	0.2
South	1.06	0.92	1.20	0.4
West	1.03	0.89	1.21	0.7
Hospital bed size				
Small	Reference			
Medium	1.04	0.87	1.23	0.7
Large	1.17	1.00	1.37	0.04
Hospital location and teaching status				
Rural	Reference			
Urban non-teaching	1.13	0.93	1.37	0.2
Urban teaching	1.23	1.02	1.48	0.03

Resource utilization in ITP hospitalizations with ICH

Table 5 shows the multivariable regression analysis assessing factors associated with LOS and hospital costs among ITP hospitalization with ICH. Overall, the LOS significantly decreased by 0.2 days per year (95% CI -0.3 to -0.1). Significantly longer LOS was noted in the age group ≥15 years (15-24 years: 1.1 days, 95% CI 0.7-1.4; 25-64 years: 2.2 days, 95% CI 2.0-2.5; ≥65 years: 2.4 days, 95% CI 2.0-2.7), with following comorbidities: GI bleed (2.5 days, 95% CI 2.1-2.9), hematuria (1.1 days, 95% CI 0.6-1.6), other bleed (1.1 days, 95% CI 0.7-1.5), among hospitalizations in the southern region (0.2 days, 95% CI 0.03-0.4), in medium (0.7 days, 95% CI 0.4-1.0) and large (0.7 days, 95% CI 0.4-1.0) size hospitals, and in urban hospitals (non-teaching: 0.7 days, 95% CI 0.4-1.0; teaching: 1.3, 95% CI 1.0-1.5). Female gender (-0.5 days, 95% CI -0.7 to -0.4), insurance type (Medicaid: -0.5 days, 95% CI -0.8 to -0.2; private: -1.3 days, 95% CI -1.5 to -1.0; self-pay/no charge/others: -1.0 days, 95% CI -1.3 to -0.6), and hospitalization in the Midwest region (-0.3 days, 95% CI -0.5 to -0.02) were associated with shorter LOS.

Significantly greater costs incurred in hospitalizations among age groups 15-24 years ($5,572, 95% CI 4,435-6,709), 25-64 years ($8,493, 95% CI 7,553-9,433), and ≥65 years ($8,504, 95% CI 7,503-9,705). Statistically significant increase in cost was also seen with comorbidities of GI bleed ($9,391, 95% CI 8,210-10,572), hematuria ($3,586, 95% CI 1,941-5,230), epistaxis ($3,286, 95% CI 1,965-4,607), and other bleeds ($5,931, 95% CI 4,565-7,296). The increase in LOS also translated to increased costs among hospitalizations at large and urban hospitals. Female gender and insurance type (Medicaid, private, self-pay/no charge/others) were associated with reduced hospital cost. No significant change in COH was noted during the study time period.

**Table 5:** Factors associated with length of stay and cost of hospitalization for Immune thrombocytopenia hospitalizations with intracranial hemorrhage.

Variable	Factors associated with length of stay among intracranial hemorrhage hospitalizations	Factors associated with cost of hospitalizations among intracranial hemorrhage hospitalizations		
	Beta coefficient (95% CI)	P-value	Beta coefficient (95% CI)	P-value
Intercept	2.6 (2.1, 3.2)	<0.001	4,324 (2,336, 6,311)	<0.001
Age in years				
0-14	Reference	Reference		
15-24	1.1 (0.7, 1.4)	<0.001	5,572 (4,435, 6,709)	<0.001
25-64	2.2 (2.0, 2.5)	<0.001	8,493 (7,553, 9,433)	<0.001
≥65	2.4 (2.0, 2.7)	<0.001	8,504 (7,503, 9,705)	<0.001
Year	-0.2 (-0.3, -0.1)	<0.001	-42 (-425, 341)	0.8
Gender				
Male	Reference	Reference		
Female	-0.5 (-0.7, -0.4)	<0.001	-3,535 (-4,028, -3,041)	<0.001
Primary payer				
Medicare	Reference	Reference		
Medicaid	-0.5 (-0.8, -0.2)	<0.001	-1,605 (-2,585, -624)	0.001
Private	-1.3 (-1.5, 1.0)	<0.001	-1,924 (-2,741, -1,107)	<0.001
Self-pay/no charge/others	-1.0 (-1.3, -0.6)	<0.001	-1,570 (-2,660, -480)	0.005
Gastrointestinal bleed	2.5 (2.1, 2.9)	<0.001	9,391 (8,210, 10,572)	<0.001
Hematuria	1.1 (0.6, 1.6)	<0.001	3,586 (1,941, 5,230)	<0.001
Epistaxis	0.3 (-0.1, 0.7)	0.1	3,286 (1,965, 4,607)	<0.001
Other bleeds	1.1 (0.7, 1.5)	<0.001	5,931 (4,565, 7,296)	<0.001
Hospital region				
Northeast	Reference	Reference		
Midwest	-0.3 (-0.5, 0.02)	0.04	-322 (-1,303, 659)	0.5
South	0.2 (0.03, 0.4)	0.035	-169 (-1,073, 735)	0.7
West	0.1 (-0.1, 0.4)	0.4	3,259 (2,230, 4,288)	<0.001
Hospital bed size				
Small	Reference	Reference		
Medium	0.7 (0.4, 1.0)	<0.0001	408 (-625, 1,440)	0.4
Large	0.7 (0.4-1.0)	<0.0001	982 (38,-481)	0.04
Hospital location and teaching status				
Rural	Reference	Reference		
Urban non-teaching	0.7 (0.4, 1.0)	<0.0001	2,859 (1,861, 3,857)	<0.001
Urban teaching	1.3 (1.0, 1.5)	<0.0001	5,427 (4,414, 6,440)	<0.001

## Discussion

Using the largest and nationally representative healthcare database in the US, we examined the trends in incidence, mortality, and resource utilization for ITP hospitalizations with ICH in the US from 2007 to 2016. Our findings indicated that the incidence of ICH in ITP hospitalizations is low. Nonetheless, it is associated with a high mortality and healthcare cost. While there was no significant change in ICH trend among ITP hospitalizations during the study period of nearly a decade, we found that increasing age was associated with higher occurrence and mortality of ICH and female gender was associated with lower occurrence and mortality. Comorbidities such as GI bleeding not only increased the likelihood of ICH and ICH-related mortality but also significantly increased LOS and COH. Our study extends the breadth of knowledge on existing data for ICH in ITP hospitalizations.

Prior literature has been conflicting when reporting the incidence of ICH among patients with ITP. This is, in part, due to the variations in study methodology. Majority of published studies have relied on either prospectively collected or registry-based data to study these outcomes [3,22]. Using prospective studies to assess this outcome could lead to potential underestimation of incidence rates, since clinical trials tend to exclude patients with prior history of severe bleeding. Additionally, due to the rarity of ICH as an outcome in patients with ITP, it is not possible to capture it as an outcome in clinical trials. Therefore, it is important to study the incidence trends and predictors of ICH through administrative claims database as done in our study, which allows a large sample size. The incidence of ICH in adult hospitalizations reported in our study is comparable to two prior studies which also used the NIS [6,9]. Danese et al. noted the incidence of 1.5% in adult hospitalizations from 2003-2006 [6]. An and Wang published outcomes of adult hospitalizations with ITP in the US from 2006 to 2012 and found the incidence of ICH at 1.28% [9]. However, unlike our study, these studies used diagnosis-related groups (DRGs) instead of ICD codes to identify the ITP hospitalizations, which may reduce their accuracy in capturing the true incidence. Tarantino et al. used the 2009 Kid’s Inpatient Database (KID) to study pediatric hospitalizations with ITP in the US [5]. The incidence of ICH was 0.6%, which is similar to our study. It is important note that none of these studies provided the incidence trends of ICH as reported in our study. Overall, the incidence in our study did not change over time in the entire population, which was reassuring given the low incidence. However, significant variations in incidence trends were noted according to patient age. Those in younger age groups (≤24 years) had declining trend and those aged ≥65 years had significant increase in trend over time. Due to the inherent limitations of the database studies, we were unable to further study the reasons for these trends. Specifically, our study lacked patient and disease-level characteristics such as time since diagnosis, platelet count preceding the event, and response to therapy which could have impacted these results. Nevertheless, these findings indicate the need for further work to study these trends in detail. While the goal of ITP treatment is to prevent major bleeding episodes, there is no evidence to show that any therapy is effective in preventing occurrence of ICH [23]. Since these studies were conducted before newer agents such as thrombopoietin receptor agonists (TPO-RA) became broadly available, it would be important to continue assessing the trends of ICH in patients with ITP to understand the impact of these medications on reducing the incidence of ICH [24,25].

Our study also assessed the risk factors for ICH. We found that increasing age was significantly associated with higher risk of ICH. Older age is a known risk factor for severe bleeding in patients with ITP [26,27]. It is likely due to the presence of comorbidities and higher usage of medications such as antiplatelet agents and anticoagulants. In a cohort of 117 patients with ITP, Cortelazzo et al. reported that the incidence of major hemorrhagic complications was significantly higher in those aged >60 years (10.4%) compared to those aged <40 years (0.4%) [26]. Similarly, Hato et al. also found that age ≥60 years was associated with higher risk of ICH in a nationwide Japanese cohort of ITP (OR 3.1, 95% CI 2.1-4.5) [27]. This study also showed that the presence of organ bleeding was associated with significantly higher risk of ICH (hematuria: OR 1.6, 95% CI 1.0-2.3). While our study did not find specific association with hematuria, GI bleeding did have an association with ICH. Similar to this study, our study also did not find association between mucosal bleeding (epistaxis) and ICH. The association with visceral bleeding is thought to be related to the possibility of profound thrombocytopenia associated with occult bleeding episodes compared to visible skin or mucosal bleeding. It is important note that our study did not have provision of platelet count data for these hospital encounters to further study the association in that context. Moreover, we found that higher age and presence of GI bleeding were also associated with higher risk of mortality. Therefore, these findings underscore the need for close monitoring and potential aggressive management instead of observation approach in older adults. Male gender was also noted to be associated with higher incidence and mortality of ICH. This finding has not been previously reported [7,8,27]. Further studies need to be conducted to understand the role of gender in occurrence of ICH. In addition, our study revealed that large urban teaching hospitals tend to have higher incidence of ICH in ITP hospitalizations compared to rural hospital setting. Although the reason for this is unclear, it is possible that this is influenced by the need to manage severe complications in large urban teaching hospitals where resources are available.

Among ITP hospitalizations, a diagnosis of ICH was associated with a significantly longer hospital stay which also translated into higher COH. These findings are similar to the An and Wang study where ICH was associated with hospital days and COH of 6.4 days (5.9-6.9) and $17,280 (15,663-18,897), respectively [9]. Similarly, Danese et al. noted the hospital stay length and cost of 6.7 days (6.2-7.3) and $14,772 (13,082-16,361), respectively [6]. Our study showed slightly lower LOS of 4.8 days (2.4-10.1); however, the COH was higher at $20,081 (9,536-41,096). The LOS discrepancy could be explained by inclusion of both adult and pediatric encounters and also by longer study time period and higher number of encounters in our study. Moreover, the cost for inflation was adjusted using 2014 figures, which could explain the higher COH shown in our study. When assessing the impact of covariates on ICH encounters, we found that increasing age and presence of comorbidities such as GI bleed and hematuria significantly increased LOS and cost. We were unable to adequately determine the specific details of the costs associated with these hospitalizations as the medication details are not adequately captured in NIS. However, these findings are possibly related to the need for aggressive clinical management for the patients with severe bleeding in ITP.

Although this study provides a national estimate of ICH incidence and mortality trends in ITP hospitalizations through a nationally representative dataset, there are certain limitations to this approach that need to be described. NIS only tracks encounter-level data and does not provide individual patient-level information. Therefore, there is a possibility of capturing multiple hospitalizations for an individual within given study timeline. Additionally, due to the lack of details on outpatient medical management or emergency room utilization which did not result in inpatient hospitalizations, we could be missing cases of ICH in this population. Since NIS data are captured through ICD codes, the data accuracy and completeness depend on accurate coding.

## Conclusions

Our study provides information on ICH epidemiology, risk factors, and outcomes which could inform clinical practice guidelines as there is an ongoing debate of “wait and watch” versus aggressive management approaches in patients with ITP. Since ICH poses a significant risk of morbidity and mortality in patients with ITP as seen in our study, early treatment of high-risk patients such as older age may be warranted to prevent devastating outcomes. Further work is also needed to study the impact of medications such as TPO-RA on mitigating these outcomes.
